# Knowledge and utilization of palliative radiotherapy by pediatric oncologists

**DOI:** 10.3747/co.v17i1.460

**Published:** 2010-02

**Authors:** T.L. Tucker, R.S. Samant, E.J. Fitzgibbon

**Affiliations:** * Division of Palliative Care, Bruyère Continuing Care, Élisabeth Bruyère Hospital, Ottawa, ON; † Faculty of Medicine, University of Ottawa, Ottawa, ON; ‡ Division of Radiation Oncology, The Ottawa Hospital Cancer Centre, Ottawa, ON; § Division of Palliative Care, The Ottawa Hospital, Ottawa, ON

**Keywords:** Pediatric oncology, palliative care, palliative radiotherapy, utilization, barriers, education, survey

## Abstract

**Background and Purpose:**

Palliative radiotherapy (prt) is a routine part of oncology care in adult patients, but it is used much less frequently among children with incurable cancer. We surveyed Canadian pediatric oncologists to learn about their knowledge and use of prt and to identify potential barriers to referral.

**Methods:**

A 13-item questionnaire assessing prt knowledge and utilization was sent to 80 Canadian pediatric oncologists.

**Results:**

The survey completion rate was 80%, with most respondents being providers of palliative care for children and making referrals for prt. Although 62% had received training in radiation oncology, only 28% had received formal palliative care training. Respondents with palliative care training were found to be significantly more knowledgeable about prt and were more likely to refer children for prt (*p* < 0.01). Only 59% of respondents thought that they had adequate knowledge about the indications for prt. A positive correlation was found between knowledge about the indications for prt and referral for treatment (*p* < 0.01). Among survey respondents, 51% believed that prt was underutilized, and the perceived barriers to prt referral included patient or family reluctance, distance to the cancer centre, belief that prt has little impact on quality of life, and concerns about toxicity.

**Conclusions:**

Palliative radiotherapy is considered to be underutilized among children. This situation appears to be related, in part, to inadequate knowledge and training among pediatric oncologists, suggesting that more emphasis needs to be placed on pediatric palliative care education.

## 1. INTRODUCTION

Radiotherapy plays a vital role in the management of many patients with cancer, in both treatment intended for cure and treatment in which the primary intent is palliation of symptoms. In fact, radiotherapy for palliative purposes can represent up to 30%–50% of the radiotherapy workload in some radiation oncology programs 1,2. As a result, radiotherapy is an essential part of any comprehensive palliative care program 3,4. But despite the growing literature to support the use of palliative radiotherapy for symptom management and quality-of-life improvement 1,5–8 in adult patients, there remains a problem of under-utilization of this treatment option 2–4,9. Several reasons for underutilization have been identified, including short life expectancy for the patient, transportation difficulties, lack of a coordinated team approach to end-of-life care, and a lack of comfort on the part of primary physicians with their radiotherapy knowledge 4,9–11.

Literature pertaining to palliative care in children with cancer is less available. Although cancer is not as prevalent in children as it is in adults, it is the second leading cause of death, after accidents, and the leading cause of death from non-accidental causes 12. Every year, approximately 850 children are diagnosed with cancer in Canada 13. Children often receive aggressive treatment until the end of life. The idea of giving palliative treatment to a child may not be welcomed by either the parents or the physicians, because the intent to cure remains a priority, and extending life may be perceived to be more important than promoting comfort 14. As well, few palliative care physicians or pediatricians in Canada have specific training and expertise in pediatric palliative care.

Although there is evidence to suggest that radiotherapy is effective in the palliation of metastasis to bone, brain, and liver, and of painful soft-tissue sites in children 15–18, information about the use of palliative radiotherapy for symptom management in children is scarce. A number of constraints may potentially limit the use of palliative radiation in children, including lack of knowledge on the part of the clinician and concerns that the treatment may be too traumatic or may not significantly contribute to the child’s quality of life.

The present study was carried out to determine the level of knowledge about the indications for, and the current utilization of, palliative radiotherapy among pediatric oncologists in Canada. Barriers to the utilization of palliative radiotherapy were explored.

## 2. MATERIALS AND METHODS

A panel consisting of a radiation oncologist (RSS) and two palliative care experts (TLT, EJF), one with a clinical background in pediatric oncology (TLT), developed a 13-item questionnaire to evaluate knowledge and utilization of palliative radiotherapy among pediatric oncologists (Appendix a).

Participants were asked questions about the number of pediatric patients seen for palliative care, the number of referrals made for palliative radiotherapy, and the indications for which patients were referred for radiotherapy. Respondents were prompted with a list of 12 potential indications from which to choose and were given the opportunity to identify others. They were asked to identify how much training they had received in palliative care and in radiation oncology.

General knowledge of palliative radiation was evaluated through correct responses to a list of 12 potential indications. Participants were asked to choose from a 4-point Likert scale including “Not effective,” “Somewhat effective,” “Very effective,” and “Don’t know.” Correct answers were determined by the radiation oncology expert (RSS) in consultation with currently available literature.

Ten perceived barriers to the use of palliative radiotherapy were listed, and participants were asked to rate how much influence each barrier had on their decision to refer for palliative radiotherapy: “Not at all,” “A little,” “Somewhat,” or “A lot.”

Participants were asked whether they felt that they had enough knowledge to identify indications for palliative radiotherapy and which harmful side effects discouraged them from referral. They were also asked to identify perceived barriers to their use of palliative radiotherapy.

Most pediatric oncology care is delivered in pediatric hospitals in Canada, and so names and addresses of potential participants were identified through telephone contact with all pediatric hospitals in the country. An initial anonymous questionnaire was sent in December 2005, and a reminder was sent in January 2006. The completed questionnaires were collected, and responses were collated. The data were analyzed using the SPSS statistical software package (version 13: SPSS, Chicago, IL, U.S.A.), and a profile of pediatric oncologists who reported referring patients for palliative radiotherapy was constructed using multivariate logistic regression modeling techniques.

## 3. RESULTS

Surveys were sent to 80 pediatric oncologists across Canada for whom addresses were available—a group that represented a majority of these specialists among the estimated 90 in the country. One returned survey was not completed and was therefore ineligible for analysis; 64 completed surveys were returned, for a survey completion rate of 80%. Most of the responding clinicians (92%) had provided palliative care for children with cancer within the preceding 12 months, and 80% had referred children for palliative radiotherapy during that time. Most of the respondents (96%) had referred 1–5 patients for palliative radiotherapy during the preceding 12 months.

### 3.1 Training

Of the survey respondents, 62% had received training in radiation oncology, at a median length of 4 weeks. Only 28% of respondents had received formal palliative care training. Previous palliative care training had a significant effect on referral rates for palliative radiotherapy: 94% of respondents who had received palliative training had made referrals, compared with 73% of respondents who had received no previous palliative training (*p* < 0.01).

### 3.2 Knowledge

Of all respondents, 59% felt that they had adequate knowledge to identify indications for palliative radiotherapy among their patients; the other 41% responded “no” or “unsure.” Of respondents who had received palliative care training, 67% were confident in their knowledge of palliative radiotherapy; only 56% of those who had not received palliative care training were confident in their knowledge (*p* < 0.05).

**FIGURE 1:**
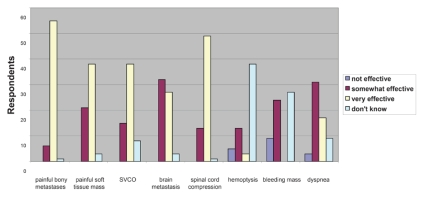
Knowledge about the effectiveness of palliative radiotherapy for various cancer-related symptoms. svco = superior vena cava obstruction.

Figure 1 shows knowledge of the effectiveness of palliative radiotherapy for specific indications. “Very effective” was considered to be correct for painful bone and soft-tissue metastasis, bleeding masses, and hemoptysis; “somewhat” or “very effective” were considered correct for the remaining indications. Knowledge of the effectiveness of palliative radiotherapy for bone metastasis, soft-tissue metastasis, and dyspnea was considered adequate in 89%, 61%, and 80% of respondents respectively. However, knowledge of palliative radiotherapy for bleeding tumour masses and hemoptysis was considered adequate in only 40% and 22% of respondents; 45% and 64% were unsure of the effectiveness of palliative radiotherapy for those indications.

Significant correlations were observed between referrals and knowledge of radiotherapy indications. In the group of respondents that made referrals for radiotherapy for bone metastasis, 98% demonstrated adequate knowledge for that indication. However, among respondents who made no referrals for bone metastasis, only 79% demonstrated adequate knowledge (*p* < 0.01). A positive correlation was also found between limited knowledge about the use of palliative radiotherapy for hemoptysis and bleeding tumour masses and the low utilization of palliative radiotherapy for those indications (*p* < 0.01).

### 3.3 Utilization

Among the 64 survey respondents, 39% had provided palliative care for 6 or more pediatric patients in the 12 months before the survey; 55% had provided palliative care for only 1–5 patients in that period. Only 4% of respondents had referred more than 5 children for palliative radiotherapy.

**FIGURE 2:**
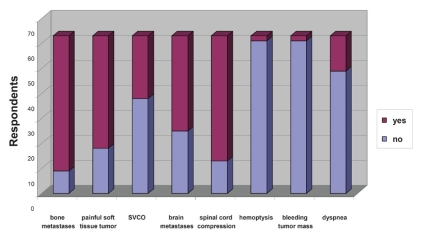
Utilization of palliative radiotherapy for various cancerrelated symptoms. svco = superior vena cava obstruction.

Respondents were asked if they had referred patients for palliative radiotherapy for each of the indications shown in Figure 2. Radiotherapy referrals were most commonly made for bone metastasis, spinal cord compression, and a painful soft-tissue mass. Only 2 respondents (3%) had referred patients for palliative radiotherapy for hemoptysis, and 2 (3%), for a bleeding tumour mass. Dyspnea because of intrathoracic tumours led to referrals by 14 respondents (22%), and 51% of respondents felt that palliative radiotherapy was underutilized in that population (19% were unsure).

When given the opportunity to comment on why they felt that radiotherapy was underutilized, most respondents offered no opinion. However, issues related to limited published literature and difficulties in coordinating treatment (especially if a radiotherapy facility was not close by) were mentioned.

### 3.4 Barriers to Utilization

**FIGURE 3:**
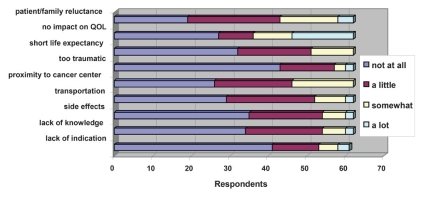
Factors influencing a decision to refer for palliative radiotherapy: perspectives from pediatric oncologists. qol = quality of life.

Of the survey respondents, 92% had access to a radiation oncologist with expertise in pediatric radiation therapy, and 94% routinely considered radiation therapy for their patients. As noted, however, approximately half (51%) the respondents felt that palliative radiotherapy is underutilized. Figure 3 outlines factors that negatively influenced the decision to refer a patient for palliative radiotherapy. Patient and family reluctance were the most prominent reasons identified, as were proximity to the cancer center and the idea that radiation treatment has little impact on quality of life.

When asked to provide details explaining previous experience with radiotherapy that discouraged referrals for palliative reasons, respondents identified nausea and vomiting that could not be well managed, fear of second malignancy, severe mucositis negatively affecting quality of life, and skin burns with ulceration and necrosis. Table I shows some of the most interesting comments by respondents about their perspectives on palliative radiotherapy.

## 4. DISCUSSION

Results from this study indicate that most pediatric oncologists in Canada perceive that they have adequate knowledge about the indications for palliative radiotherapy; however, at least half feel that palliative radiotherapy is underutilized. The exact reasons for this perceived underutilization remain unclear. Published studies 4,9–11 have attributed this radiotherapy underutilization to a number of factors (Table II). Interestingly, many of the same barriers or concerns were reported by our respondents. In the pediatric population, as in the adult population, many of these issues can and should be addressed using initiatives such as improved education and training, better communication with patients and families, and (among health care professionals) better coordination of care (to reduce patient wait times and inconvenience) and development of treatment guidelines. Given that ample evidence shows that palliative radiotherapy can effectively and fairly quickly improve symptoms and quality of life with relatively little morbidity, and often with very short courses of treatment, radiotherapy efficacy and toxicity should not be a major issue. Canada’s universal health care coverage means that radiotherapy costs are not an issue for patients, and it has even been suggested that increased use of palliative radiotherapy could not only increase quality of life for patients, but actually reduce overall costs for end-of-life care 4.

This retrospective study could not evaluate true palliative radiotherapy utilization rates, but the perception that palliative radiotherapy is underutilized may be related to limited training for most pediatric oncologists in palliative care and palliative radiotherapy. Many oncologists treating adults have also been found to have limited palliative care training, and better education regarding pain and symptom management for end-of-life care has been suggested 9. Some of our survey respondents indicated that pediatric oncologists are reluctant to accept their patients as “palliative” and delay referral for palliative radiotherapy for that reason.

Our study also noted that utilization of palliative radiotherapy was particularly low for indications such as hemoptysis and bleeding tumours. This low referral rate may in part result from a lack of knowledge or a lower prevalence of these symptoms in the pediatric oncology population. With a lack of pertinent literature, the prevalence of these symptoms in the pediatric population is largely unknown, and the role of radiotherapy is not entirely clear. However, there is no reason to believe that palliative radiotherapy would be any less effective in children than it is in adults. The adult literature 9,10 also suggests that, although bone and brain metastases and malignant spinal cord compression are well-known indications for palliative radiotherapy, the value of radiotherapy for managing other symptoms—including hemoptysis and bleeding, and painful soft-tissue lesions—is less well known among non-radiation oncologists. Our results among pediatric oncologists appear consistent with those findings.

As identified by our respondents, the most significant barriers to the utilization of palliative radiotherapy involve patient and family factors, especially reluctance on the part of patients and families to engage in palliative radiotherapy. Experience in the adult palliative care population suggests that patients are sometimes reluctant to consider palliative radiotherapy because they do not want to spend precious remaining time receiving treatments or they do not understand the role of palliative radiotherapy for symptom relief regardless of the incurable nature of the tumour. Careful explanation of the fewer treatments and relatively little toxicity 1,19,20 can sometimes alleviate reluctance and encourage patients to consider palliative treatment. Whether a similar reluctance plays a role in the pediatric cancer population is unclear. However, our study highlights a need that has been identified before 9 for radiation oncologists to be more involved in end-of-life issues for cancer patients and to work collaboratively with the pediatric oncology health care team to be able to offer palliative radiotherapy in a timely, efficient, and effective manner. Radiation oncologists dealing with pediatric patients clearly need to become more involved with symptom management for children with incurable cancers, and both ongoing research into, and development of guidelines for, the use of palliative radiotherapy in the pediatric oncology population would be helpful.

Not surprisingly, clinicians with past training in and greater knowledge of palliative care are more likely to refer for palliative radiotherapy. Although skills and knowledge in palliative care are key parts of caring for oncology patients, most of our respondents had not received formal training in palliative care. Training and education in palliative care are lacking in most pediatric residency programs 21 despite the fact that such education can enhance the palliative care knowledge of learners in those programs 22. Continuing postgraduate medical education for practicing pediatric oncologists should also highlight the importance of palliative care and palliative radiotherapy education, ensuring that this knowledge is considered an essential aspect of caring for children with cancer. The number of children dying from cancer is low; however, pediatric oncology training programs need to emphasize the effect of palliative care on symptom control for all children with cancer. Improved education in palliative care and palliative radiotherapy may increase referral rates in the pediatric patient population and may increase the overall quality of care.

Concerns remain that fear of radiation side effects may discourage oncologists from referring for radiotherapy. Some of the reasons cited, such as second malignancy, are not relevant in the non-curative setting, especially given that patients are unlikely to survive long enough to develop a second malignancy (a side effect that tends to occur only one or more decades later). Other radiation side effects such as nausea, vomiting, and mucositis are often less problematic in a palliative population, because the doses are smaller than in curative setting, and the side effects that do occur can usually be prevented or treated effectively with relatively simple approaches 1,6,23,24. It would be important in future studies to determine which side effects of radiotherapy are suffered by children receiving palliative doses and how well those side effects are managed.

Our study has several limitations. First, the retrospective design inherent in survey evaluations means that physician recall may be unreliable; actual patient referrals for palliative radiotherapy could not be independently verified. Although palliative radiotherapy was defined in the questionnaire, respondents may have varied in their own conception of palliative radiotherapy, and they may have varied in their conception of which patients were palliative. Also, the questions evaluating palliative radiotherapy indications were not exhaustive or validated, and they may not have accurately reflected palliative radiotherapy knowledge. However, the goal here was simply to assess whether common indications for palliative radiotherapy were known, rather than to fully assess knowledge, and the results were entirely consistent with published studies looking at physicians caring for adult oncology patients. Also, because our response rate was high, and because most of the pediatric oncologists in the country were represented, we believe that our results likely provide an adequate reflection of current knowledge about indications for referral.

## 5. CONCLUSIONS

Our study highlights some of the gaps in knowledge about palliative radiotherapy among pediatric oncologists in Canada and about the perceived underutilization of this treatment modality. Greater involvement by radiation oncologists is needed in pediatric palliative care, as is development of guidelines and educational initiatives that improve palliative care teaching for trainees in pediatric oncology fellowship programs and that place more emphasis on palliative radiotherapy. Initiatives of that kind may increase utilization of palliative radiotherapy, and in turn, lead to improved symptom management for children with advanced and incurable cancers.

## Figures and Tables

**TABLE I:** Comments of pediatric oncologists regarding palliative radiotherapy

“Pediatric oncologists … are somewhat reluctant to accept the inevitable outcome in some cases, and referral is delayed, causing delay in palliative radiotherapy.”
“It’s easy to contemplate radiotherapy if you’re in a centre that can provide it…. If you need to travel, this can be much more difficult to arrange and support.”
“I feel that a broader knowledge of the potential benefits of radiation for these children would improve qol.”
“It will be excellent … to produce Canadian guidelines where palliative radiotherapy in children will be a valid consideration.”

**TABLE II:** Barriers to palliative radiotherapy use based on published studies 4–6,18

Lack of education or deficiencies in training
Perceived patient and family reluctance
Distance from radiotherapy facility or transportation difficulties
Concerns about the efficacy of radiotherapy and delayed onset of symptom relief
Concerns about potential radiation toxicity
Short patient life expectancy and poor patient performance status
Perceived protracted course of treatment
Patient age (older patients are usually less likely to be referred among adult population)
Waiting times for assessment and treatment by radiation oncologists
Poor communication with oncologists
Lack of coordinated end-of-life care
Lack of treatment guidelines
Costs associated with palliative radiotherapy treatment

## APPENDIX A

### Survey Questionnaire

Palliative radiotherapy for symptom management in palliative paediatric oncology patients in Canada.

* Definition of palliative radiotherapy: Radiation therapy which is not considered curative and in which the primary intent is symptom relief.

#### A. Background Information

1. Which of the following best describes your role?
  - Paediatric Oncologist and/or Paediatric Haematologist
  - Radiation Oncologist
  - Palliative Care Physician with training in adult palliative care only
  - Palliative Care Physician with training in pediatric palliative care
  - Paediatric Haem/Onc Fellow
  - Paediatrician
  - Anaesthetist
  - Nurse
  - Family Physician
2. How many children have you seen for palliative care in the last 12 months?
  - None
  - 1–5
  - 6–10
  - 11–20
  - >20
3. How many children have you referred for assessment for palliative radiotherapy in the past 12 months?
  - None
  - 1–5
  - 6–10
  - >10
4. Have you received palliative care training?
  - Yes
  - No
  If yes, please indicate the number of weeks: __________ weeks
  Please indicate the format:
  - certified training in a palliative medicine fellowship
  - rotation in a training program other than palliative medicine
  - workshops/lectures
  - other: ________________________
5. Have you received radiation oncology experience in your training? *(If you are a Radiation Oncologist, skip to question 8)*
  - Yes
  - No
  If yes, please indicate the number of weeks:
  __________ weeks
6. Do you have access to a Radiation Oncologist with expertise in Paediatric Radiotherapy?
  - yes
  - no
  - unsure
7. Do you routinely consider radiotherapy as an option for the palliation or symptom control of your pediatric patients with cancer?
  - Yes
  - No
  - Never
  If no or never, please specify why not.
  ___________________________________
  ___________________________________
  ___________________________________
  ___________________________________
  ___________________________________
8. Please identify any of the following indications for which you have requested palliative radiotherapy for children in the past, or for which you have treated a child.
  - bone metastasis
  - impending fracture
  - painful soft tissue tumor
  - chest pain with lung mass
  - superior vena cave obstruction
  - brain metastasis
  - spinal cord compression
  - tumor induced hemoptysis
  - splenomegaly
  - hepatomegaly
  - bleeding of a tumor mass
  - shortness of breath due to intrathoracic tumor
  - other ___________________________

#### B. Knowledge of Palliative Radiotherapy

9. How effective do you consider radiotherapy for the treatment of the following cancer related symptoms? *Please* ***circle*** *the most appropriate response.*
  **Table:**
  	Not effective	Somewhat effective	Very effective	Don’t know
  a. Painful bony metastases	1	2	3	4
  b. Pathological/impending fracture	1	2	3	4
  c. Painful soft tissue tumour mass	1	2	3	4
  d. Chest pain with lung mass	1	2	3	4
  e. Superior vena cava obstruction	1	2	3	4
  f. Brain metastasis	1	2	3	4
  g. Spinal cord compression	1	2	3	4
  h. Shortness of breath due to intrathoracic tumour	1	2	3	4
  i. Tumour induced hemoptysis	1	2	3	4
  j. Splenomegaly	1	2	3	4
  k. Bleeding from a tumour mass	1	2	3	4
  l. Hepatomegaly	1	2	3	4
10. Please review the following potential barriers to your consideration of radiotherapy in children with cancer. Indicate how much each factor influences your decision.
  **Table:**
  	Not at all	A little	Somewhat	A lot
  Lack of indication in children	1	2	3	4
  Personal lack of knowledge re: indications and use of palliative radiation in children	1	2	3	4
  Concern regarding severity and duration of side effects	1	2	3	4
  Transportation difficulties	1	2	3	4
  Distance child lives from cancer center	1	2	3	4
  Too traumatic for patient and family	1	2	3	4
  Life expectancy of the child too short	1	2	3	4
  Treatment results will not impact on quality of life of the child	1	2	3	4
  No specific Paediatric Radiation Oncology expertise locally available	1	2	3	4
  Patient or family reluctance	1	2	3	4
11. Do you feel that you have adequate knowledge to identify indications for palliative radiotherapy treatment in children?
  - Yes
  - No
  - Unsure
12. Do you consider that radiotherapy is underutilized as a treatment modality in palliation for children with cancer?
  - Yes
  - No
  - Unsure
  Comments
  ___________________________________
  ___________________________________
  ____________________________________
13. Have you seen harmful side effects or sequelae of radiotherapy that have discouraged you from its use?
  - Yes
  - No
  Please explain:
  ___________________________________
  ___________________________________
  ___________________________________

THANK YOU for your assistance! Other comments?

_________________________________________

_________________________________________

_________________________________________

## References

[1] Hoegler D Radiotherapy for palliation of symptoms in incurable cancer Curr Probl Cancer 1997 21 129 83 9202888 10.1016/s0147-0272(97)80004-9

[2] Janjan NA An emerging respect for palliative care in radiation oncology J Palliat Med 1998 1 83 8 15859875 10.1089/jpm.1998.1.83

[3] Guang J Zhou S Groome P Tyldesley S Zhang–Solomons J Mackillop WJ Factors affecting the use of palliative radiotherapy in Ontario J Clin Oncol 2001 19 137 44 11134206 10.1200/JCO.2001.19.1.137

[4] Skolnick AA New study suggests radiation often underused for palliation JAMA 1998 279 343 4 9459453 10.1001/jama.279.5.343

[5] Ashby M The role of radiotherapy in palliative care J Pain Symptom Manage 1991 6 380 8 1715370 10.1016/0885-3924(91)90030-8

[6] Ciezki JP Komurcu S Macklis RM Palliative radiotherapy Semin Oncol 2000 27 90 3 10697025

[7] Donato V Bonfili P Bulzonetti N Radiation therapy for the oncological emergences Anticancer Res 2001 21 2219 24 11501850

[8] Wu JS Monk G Clark T Robinson J Eigl BJ Hagen N Palliative radiotherapy improves pain and reduces functional interference in patients with painful bone metastases: a quality assurance study Clin Oncol (R Coll Radiol) 2006 18 539 44 16969984 10.1016/j.clon.2006.05.003

[9] McCloskey SA Tao ML Rose CM Fink A Amadeo AM National survey of perspectives of palliative radiation therapy: role, barriers, and needs Cancer J 2007 13 130 7 17476142 10.1097/PPO.0b013e31804675d4

[10] Barnes EA Parliament M Hanson J Watanabe S Palmer JL Bruera E Palliative radiotherapy for patients with bone metastases: survey of primary care physicians Radiother Oncol 2003 67 221 3 12812854 10.1016/s0167-8140(02)00366-3

[11] Lutz S Spence C Chow E Janjan N Connor S Survey on the use of palliative radiotherapy in hospice care J Clin Oncol 2004 22 3581 6 15337808 10.1200/JCO.2004.11.151

[12] Arias E MacDorman MF Strobino DM Guyer B Annual summary of vital statistics—2002 Pediatrics 2003 112 1215 30 14654589 10.1542/peds.112.6.1215

[13] Canadian Cancer Society and the National Cancer Institute of Canada Canadian Cancer Statistics 2008 Toronto Canadian Cancer Society 2008

[14] Himelstein BP Hilden JM Boldt AM Weissman D Pediatric palliative care N Engl J Med 2004 350 1752 62 15103002 10.1056/NEJMra030334

[15] Deutsch M Tersak JM Radiotherapy for symptomatic metastases to bone in children Am J Clin Oncol 2004 27 128 31 15057150 10.1097/01.coc.0000046807.66194.a5

[16] Koontz BF Clough RW Halperin EC Palliative radiation therapy for metastatic Ewing sarcoma Cancer 2006 106 1790 3 16534788 10.1002/cncr.21812

[17] Paulino AC Relapsed Wilms tumor: is there a role for radiation therapy? Am J Clin Oncol 2001 24 408 13 11474275 10.1097/00000421-200108000-00022

[18] Paulino AC Palliative radiotherapy in children with neuroblastoma Pediatr Hematol Oncol 2003 20 111 17 12554522 10.1080/0880010390158702

[19] Plataniotis GA Kouvaris JR Dardoufas C Kouloulias V Theofanopoulou MA Vlahos L A short radiotherapy course for locally advanced non-small cell lung cancer (nsclc): effective palliation and patients’ convenience Lung Cancer 2002 35 203 7 11804694 10.1016/s0169-5002(01)00327-0

[20] Shakespeare TP Lu JJ Back MF Liang S Mukherjee RK Wynne CJ Patient preference for radiotherapy fractionation schedule in the palliation of painful bone metastases J Clin Oncol 2003 21 2156 62 12775741 10.1200/JCO.2003.10.112

[21] Baker JN Torkildson C Baillargeon JG Olney CA Kane JR National survey of pediatric residency program directors and residents regarding education in palliative medicine and end-of-life care J Palliat Med 2007 10 420 9 17472514 10.1089/jpm.2006.0135

[22] Baughcum AE Gerhardt CA Young–Saleme T Stefanik R Klopfenstein KJ Evaluation of a pediatric palliative care educational workshop for oncology fellows Pediatr Blood Cancer 2007 49 154 9 16991132 10.1002/pbc.21034

[23] Samant R Gooi AC Radiotherapy basics for family physicians. Potent tool for symptom relief Can Fam Physician 2005 51 1496 501 16353832 PMC1479480

[24] Tisdale BA When to consider radiation therapy for your patient Am Fam Physician 1999 59 1177 84 10088874

